# Trastorno del Neurodesarrollo con Movimientos Anormales Debido a Mutación en el Gen *GNAO1*: A Propósito de un Caso

**DOI:** 10.31083/RN46799

**Published:** 2025-09-22

**Authors:** Sandra Milena Hernández Yeneris, María Alejandra González-Solano, Isabella Lince-Rivera, Jorge A. Rojas-Martínez, Jorge L. Ramón-Gómez

**Affiliations:** ^1^Servicio de Pediatría, Hospital Universitario San Ignacio e Instituto Roosevelt-Pontificia Universidad Javeriana, 110231 Bogotá, Colombia; ^2^Servicio de Neurología Pediátrica, Universidad Militar Nueva Granada, 110231 Bogotá, Colombia; ^3^Servicio de Genética, Clínica del Country, 110221 Bogotá, Colombia; ^4^Servicio de Neurología Pediátrica, Instituto Roosevelt, 110321 Bogotá, Colombia

**Keywords:** movimientos anormales, gen *GNAO1*, mutación, neurodesarrollo, trastornos neurológicos, abnormal movements, *GNAO1* gene, mutation, neurodevelopment, neurological disorders

## Abstract

**Introducción:**

Presentamos el caso de una paciente con una variante probablemente patógena heterocigota *de novo* c.545C>T (p.Thr182Ile) en el gen *GNAO1* en relación con un trastorno del neurodesarrollo con movimientos anormales.

**Caso Clínico:**

Niña que desde los 3 meses presentaba retraso grave del neurodesarrollo, y posteriormente asoció mioclonías, discinesias orofaciales y coreoatetosis, sin crisis ictales. Se le realizaron estudios que descartaron causas de origen metabólico y estructural, y finalmente se le realizó secuenciación exómica completa en trío que informó de una variante probablemente patógena heterocigota *de novo*, c.545C>T (p.Thr182Ile), en el gen *GNAO1*.

**Conclusiones:**

El reconocimiento temprano del retraso en el neurodesarrollo y los movimientos anormales son determinantes para el enfoque etiológico de un trastorno neurológico, y se debe promover el uso de la secuenciación completa del exoma si se ha descartado un diagnóstico estructural y metabólico, dado que la identificación de una condición específica repercute en el tratamiento, el pronóstico y el asesoramiento genético.

## Abstract (en)

**Introduction:**

We present the case of a patient with a *de novo* heterozygous probably pathogenic variant c.545C>T (p.Thr182Ile) in the *GNAO1* gene that is probably pathogenic in relation to a neurodevelopmental disorder and movement disorder.

**Case Report:**

A female patient who started at 3 months with severe neurodevelopmental delay, and subsequently myoclonus, orofacial dyskinesia, and choreoathetosis, without seizures. Metabolic and structural causes were investigated and, finally, whole exome sequencing in trio identified a *de novo* heterozygous, probably pathogenic, variant c.545C>T (p.Thr182Ile) in the *GNAO1* gene.

**Conclusions:**

Early recognition of neurodevelopmental delay and abnormal movements are determinants of an etiological approach to a neurological disorder. The use of whole exome sequencing should be promoted if a structural and metabolic diagnosis has been ruled out as the identification of a specific condition affects its management and prognosis, and guides genetic counseling.

## 1. Introducción

El gen *GNAO1*, localizado en el cromosoma 16q13, codifica la subunidad alfa de las proteínas G, que son complejos heterotriméricos transductores de señales altamente expresados en el sistema nervioso central, con un papel importante en la modulación de la neurotransmisión y la excitabilidad neuronal [1, 2]. Las mutaciones en este gen causan un espectro complejo de patologías neurológicas, que incluyen epilepsia, retraso en el neurodesarrollo y trastornos del movimiento [3, 4].

Se ha descrito que las variantes de pérdida de función en este gen están principalmente asociadas con encefalopatía epiléptica, y las de ganancia de función con trastornos del movimiento [2]. Sin embargo, el espectro parece ser más amplio, y no sólo existe variabilidad en la presentación fenotípica, sino que la gravedad también parece estar influida por la variante *per se* y su efecto en la función de la proteína G, como se ha estudiado recientemente [5].

Las manifestaciones clínicas son variables y se caracterizan por trastornos del movimiento, retraso del neurodesarrollo, déficit cognitivo y diferentes tipos de epilepsia, y es frecuente su refractariedad [3]. La enfermedad generalmente comienza con síntomas inespecíficos, como hipotonía y alteración del perímetro cefálico, y evoluciona con el tiempo, simulando otras causas de trastornos del neurodesarrollo y dificultando el reconocimiento temprano. En la mayoría de los casos se presenta antes del año de vida, y es más frecuente en los menores de 6 meses [4, 6].

Respecto a los estudios paraclínicos, el electroencefalograma puede evidenciar un patrón de estallido-supresión, hipsarritmia, descargas multifocales y/o enlentecimiento de la actividad de fondo, y las neuroimágenes se caracterizan por mostrar una atrofia cerebral progresiva, retraso en la mielinización y/o disgenesia del cuerpo calloso [7].

No se ha identificado un medicamento único efectivo ni existe actualmente una terapia modificadora de la enfermedad. Se ha descrito la respuesta farmacológica como incompleta [8]; sin embargo, el uso de benzodiacepinas es frecuente [6] y se ha comunicado la efectividad de la dieta cetógena, la tetrabenazine, el levetiracetam, el topiramato y el trihexifenidilo en algunos casos para reducir o eliminar movimientos hipercinéticos [2, 9].

A continuación, se describe el caso de una paciente con un trastorno del movimiento progresivo, con una variante probablemente patógena heterocigota *de novo*, c. 545C>T (p.Thr182lle), en el gen *GNAO1*, lo cual añade evidencia al espectro fenotípico notificado. La presente investigación sigue la lista de verificación CARE checklist (**Material Suplementario-CARE-checklist-Spanish-2013**).

## 2. Caso Clínico

Se trata de una paciente femenina fruto de un embarazo controlado, con ecografías y paraclínicos infecciosos prenatales normales, sin exposición a teratógenos, padres no consanguíneos, nacida a las 39 semanas vía cesárea por iterativa, antropometría adecuada para la edad y adaptación neonatal espontánea. Como antecedentes familiares, un hermano falleció a los 8 días de vida por hernia diafragmática congénita y el padre padece lupus eritematoso sistémico, hipertensión arterial y un tromboembolismo pulmonar.

Se le valoró por genética a los 10 meses por un cuadro que comenzó desde los 3 meses, consistente en retraso del neurodesarrollo. Durante la valoración se evidenciaron anomalías menores, sostén cefálico y rolados incompletos, sin agarre de objetos, sin seguimiento visual, escasa interacción con el examinador, hipotonía axial, hipertonía apendicular e hiperreflexia generalizada. Había realizado fisioterapia ambulatoria con escasos avances, por lo cual, junto con neurología pediátrica, se consideró realizar estudios.

Se solicitó piruvato, lactato, amonio, creatincinasa, perfil tiroideo, transaminasas, deshidrogenasa láctica y azoados, que resultaron normales, y hemograma, con trombocitosis leve. La resonancia magnética cerebral simple, el videoelectroencefalograma, el ecocardiograma y la videocinedeglución resultaron sin alteraciones. Se le valoró por oftalmología, con un examen normal. Se realizó cribado para la enfermedad de Pompe, con valores enzimáticos de la alfa-glucosidasa comparables a controles normales de población colombiana, cariotipo con resultado 46,XX, aminoácidos cuantitativos en el plasma e hibridación genómica comparada por micromatrices, todos con resultados normales.

Asistió a control a los 15 meses con los resultados mencionados, sin progreso en los hitos del desarrollo. Se realizó secuenciación exómica completa en trío con evidencia de una variante probablemente patógena heterocigota c.545C>T (p.T182I) en el gen *GNAO1* (OMIM *139311), cuya frecuencia alélica es desconocida, ya que no se ha identificado en alelos en bases de datos poblacionales (gnomAD, http://gnomad.broadinstitute.org; base de datos de polimorfismos de nucleótido único (dbSNP) rs1596871452), y tiene una sola entrada en la base de datos ClinVar (revisado el 1 de abril de 2024) como probablemente patógena. En conclusión, la variante p.Thr182Ile cumple los criterios del American College of Medical Genetics and Genomics para ser clasificada como una variante probablemente patógena para trastornos relacionados con el gen *GNAO1*. Esta variante, según el análisis *in silico*, se localiza en la región Swicth 1 de la proteína, por lo que llevaría a una disminución significativa en la formación de heterotrímeros. Todos los predictores *in silico* utilizados, tanto los MetaScores (BayesDel addAF, BayesDel noAF, MetaRNN y REVEL) como los individuales (EIGEN, MutPred, PrimateAI, PROVEAN, SIFT, MutationTaster y PolyPhen-2), indican que es deletérea. Adicionalmente, la secuenciación del exoma completo no identificó esta variante en los padres, por lo cual se consideró una variante *de novo*.

Durante los controles la madre refirió movimientos anormales compatibles con mioclonías y en el examen físico se evidenció tono fluctuante en las extremidades, impresionando posturas distónicas. Se consideró trastorno del neurodesarrollo con movimientos anormales (OMIM #617493) de herencia autosómica dominante secundario a la variante identificada, y se inició topiramato.

A los 23 meses se documentó microcefalia, plagiocefalia, discinesias orofaciales, mioclonías y movimientos coreiformes en los miembros superiores, e hipotonía generalizada de predominio axial. Continuó el tratamiento multidisciplinario y las terapias, con lo cual logró avances en algunos hitos del neurodesarrollo. La presencia de mioclonías llevó a una videotelemetría de control (que descartó una etiología ictal) y a un aumento en la dosis del topiramato hasta 5 mg/kg/día, con mejoría de aquéllas y respuesta parcial de las discinesias orofaciales.

A los 29 meses se documentó reaparición de las mioclonías, corea y distonía, sin crisis epilépticas. Se decidió suspender el topiramato e iniciar tetrabenazine de manera progresiva hasta 3 mg/kg/día, sin respuesta. Ante el aumento de la distonía, de predominio axial, y el alto riesgo de presentar estado distónico, se añadió clonazepam, en dosis de 0,03 mg/kg/día.

Posteriormente se le hospitalizó por infección respiratoria aguda que facilitó un estado convulsivo distónico y el posterior fallecimiento de la paciente en el contexto de una infección concurrente, ya conocido previamente como un disparador común del estado.

## 3. Discusión

En 2013 se describieron por primera vez las mutaciones del gen *GNAO1* en pacientes con epilepsia de aparición temprana. Hasta la fecha se han descrito dos fenotipos: encefalopatía epiléptica grave de inicio temprano y trastorno del neurodesarrollo con movimientos anormales, con o sin crisis epilépticas [3, 4]. La presencia de fenotipos intermedios dentro del espectro de la condición es un fenómeno cada vez más reconocido, con pacientes que muestran una mezcla de epilepsia y trastorno del movimiento, de gravedad variable [10].

Para la confirmación diagnóstica se requieren estudios moleculares [7]. Todos los casos notificados hasta el momento son únicos, producto de mutaciones *de novo.* Es decir, a pesar de la herencia autosómica dominante descrita, ningún paciente afectado ha tenido descendencia. El asesoramiento genético es muy valioso para informar a los padres de que el riesgo de tener más hijos afectados es bajo.

En el caso presentado encontramos una paciente con inicio de sintomatología inespecífica, retraso en el neurodesarrollo, trastorno del movimiento hipercinético, mioclonías, distonía y movimientos coreiformes, estudios metabólicos negativos y una secuenciación completa del exoma con evidencia de una variante probablemente patógena heterocigota *de novo*, c.545C>T (T182I), en el gen *GNAO1*, lo que confirma el diagnóstico de un trastorno neurológico asociado a *GNAO1*. La variante T182I ha sido descrita previamente en la bibliografía [5], y el fenotipo involucraba la presencia de un cuadro grave, afectación del neurodesarrollo y trastorno del movimiento con coreodistonía y crisis discinéticas.

El trastorno del movimiento usualmente comienza en la infancia, y puede constar de coreoatetosis, balismo, mioclonías, distonía, ataxia, estereotipias manuales y/o discinesias orofaciales, con exacerbaciones recurrentes que pueden ser desencadenadas por factores estresantes y que pueden llevar a estado distónico [3, 8]. La mayoría de estos pacientes presenta discapacidad cognitiva y motora marcada, aunque recientemente se han notificado variantes asociadas a fenotipos leves [3, 6].

El tratamiento temprano y adecuado de los movimientos anormales y el seguimiento de la respuesta terapéutica vigilando la aparición de complicaciones son fundamentales. Usualmente, el tratamiento es desafiante y requiere la combinación de varios medicamentos, y la tetrabenazine es la más efectiva para el control de la corea [8, 11]. El uso de benzodiacepinas y fármacos anticrisis es frecuente, aunque su efectividad es menor [11]. Las opciones de tratamiento farmacológico y sus dosis están descritas en la Tabla 1 [12, 13, 14], sin dejar a un lado que la estimulación cerebral profunda con el globo pálido interno como objetivo y la palidotomía también han conseguido efectividad en esta entidad [15].

**Tabla 1.** **Opciones de manejo farmacológico descritas en movimientos anormales debidos a mutación en el gen *GNAO1* (Tomado de [12, 13, 14])**.

	Mecanismo de acción	Dosis	Efectos adversos
tetrabenazine	Acción sobre VMAT2: depleta múltiples neurotransmisores de amina (dopamina, norepinefrina, serotonina)	Inicial de 6,5–12,5 mg/día, aumento de 12,5 mg/día cada 3–5 días hasta 50 mg/día dividido en tres dosis	Parkinsonismo, somnolencia, sedación, acatisia, insomnio
Trihexifenidilo	Antagonista selectivo del receptor muscarínico M1	Inicial a 0,2 mg/kg/día dividido en tres dosis, aumento semanal de la misma dosis hasta 2 mg/kg	Estreñimiento, boca seca
clonazepam	Agonista del GABA	Inicial 0,25 mg, aumento de 0,25–0,5 mg/día cada 3–4 días hasta 0,1–0,2 mg/kg/día dividido en 2–3 dosis	Sedación, aumento de secreciones, confusión, psicosis
diazepam	Agonista del GABA	0,2 mg/kg/dosis tres dosis al día	Sedación, aumento de secreciones, confusión
Clonidina	Agonista central alfa_2_-adrenérgico	3–5 µg/kg/día dividido en tres dosis	Somnolencia, mareo, boca seca, estreñimiento, cefalea
Topiramato	Mixto (bloqueo de los canales de sodio, calcio, AMPA, aumento del GABA, inhibición anhidrasa carbónica)	Inicial 25 mg/día, aumento a 5–10 mg/kg/día dividido en dos dosis	Disminución de la velocidad de procesamiento, alteraciones en la atención y la memoria, parestesias, anorexia, litiasis
Levetiracetam	Unión a SV2A de vesícula sináptica para inhibir de forma global la liberación de neurotransmisores	Inicial a 15–20 mg/kg/día, aumento hasta 60 mg/kg/día dividido en dos dosis	Somnolencia, mareo, labilidad emocional, agresividad, depresión, psicosis
Carbamacepina	Bloqueo de los canales de sodio dependientes del voltaje	Inicial de 7–10 mg/kg/día hasta 20–30 mg/kg/día dividido en tres dosis	Mareo, náuseas, somnolencia, fatiga, visión borrosa, hiponatremia, cefalea, supresión médula ósea, exantema
Ácido valproico	Mixto (bloqueo de los canales de sodio, calcio, aumento del GABA)	Inicial de 10–15 mg/kg/día dividido en dos dosis, aumento de 5–10 mg/kg cada semana hasta 25–30 mg/kg/día	Náuseas, emesis, ganancia de peso, hiperamonemia, lesión hepática, alopecia, trombocitopenia, resistencia a la insulina
Levodopa	Precursor de dopamina	Inicial 1 mg/kg/día hasta 4–5 mg/kg/día dividido en tres dosis	Náuseas, mareo, alucinaciones
Haloperidol	Bloqueo de los receptores dopaminérgicos D2 postsinápticos	Inicial de 0,25–0,5 mg/kg y aumentar 0,25–0,5 mg cada semana dividido en 1–2 dosis	Distonía, acatisia, temblores, síndrome neuroléptico maligno, parkinsonismo
Risperidona	Antagonista de los receptores de la serotonina 5-HT y la dopamina D2	Inicial de 0,25 o 0,5 mg/día hasta 2–4 mg/día dividido en dos dosis	Hipotensión, somnolencia, aumento de peso, hiperglucemia, acatisia, parkinsonismo
Gabapentina	Inhibición de los canales de calcio dependientes del voltaje	Inicial de 10–15 mg/kg/día hasta 50–300 mg/día dividido en tres dosis	Sedación, mareo, aumento de peso, cambios visuales

AMPA, α-amino-3-hidroxi-5-metilo-4-isoxazolpropiónico; GABA, ácido gamma aminobutírico; VMAT2, transportador de monoaminas vesicular tipo 2; AMPA, ácido alfa-amino-3-hidroxi-5-metil-4-isoxazol propiónico; SV2A, proteína 2A de la vesícula sináptica; 5-HT, 5-hidroxitriptamina.

## 4. Conclusiones

Los trastornos neurológicos asociados a *GNAO1* representan un reto diagnóstico, dada la escasa bibliografía disponible al respecto y el espectro fenotípico amplio, y se pueden presentar con síntomas aislados o inespecíficos, como hipotonía. Éste es uno de los pocos casos de pacientes con mutación en *GNAO1* asociada a un cuadro neurológico grave notificado en Colombia, y pone en manifiesto la importancia de usar herramientas diagnósticas, como la secuenciación completa del exoma, en niños con retraso del neurodesarrollo cuando se ha descartado un diagnóstico metabólico u otra patología orgánica, pues la identificación de mutaciones en genes como éste repercute en el tratamiento, el pronóstico y el asesoramiento genético del paciente y su familia.

## Data Availability

El intercambio de datos no es aplicable, ya que no se generaron ni analizaron datos.

## Contribuciones de los Autores

SMHY, MAGS, ILR, JRM and JRG: concepción/diseño, análisis de datos e interpretación, redacción/revisión del artículo. Todos los autores han leído y aprobado la versión final del manuscrito. Todos los autores han participado plenamente en este trabajo y están de acuerdo en asumir la responsabilidad de todos los aspectos de este trabajo.

## Aprobación Ética y Consentimiento Informado

La publicación de este reporte de caso se llevó a cabo respetando los principios éticos y las normativas vigentes para la divulgación de información médica. Se obtuvo el consentimiento informado por escrito del tutor legal del paciente. Además, el estudio cumple con los lineamientos de la Declaración de Helsinki y otras normativas internacionales sobre ética en la publicación de casos clínicos. Este caso fue evaluado y aprobado por el Comité de Investigaciones y Ética Institucional (CIEI) de la Facultad de Medicina Pontificia Universidad Javeriana-Hospital Universitario San Ignacio antes de su publicación, con número de acta (01/2025).

## Agradecimientos

No aplicable.

## Financiación

Esta investigación no recibió financiación externa.

## Material Suplementario

El material suplementario asociado con este artículo se puede encontrar, en la versión en línea, en https://doi.org/10.31083/RN46799.
