# Supplementary material for: Neurodevelopmental and Movement Disorder Due to a Mutation in the GNAO1 Gene: A Case Report
Source: Rev Neurol. 2025 Sep 22;80(8):46799. [Article in Spanish] doi: 10.31083/RN46799 (PMC12516824; doi:10.31083/RN46799)
Supplement: Supplementary file 1 [file 1576-6578-80-8-46799-s1.zip › Material Suplementario-CARE-checklist-Spanish-2013.pdf]

CARE case report guidelines

Lista de comprobación CARE (2013) de la información a incluir al Redactar un informe de caso

(cc) BY-NC-ND

Asunto	Elemento	Descripción del elemento de la lista de comprobación	Informado en la página
**Título**	**1**	Las palabras "informe de caso" deben aparecer en el título junto con lo más interesante de este caso.	1
**Palabras clave**	2	Los elementos clave de este caso en 2 - 5 palabras clave.	1
**Resumen**	**3a**	Introducción— ¿Qué es único en este caso? ¿Qué aporta de nuevo a la literatura médica?	1
	**3b**	Los principales síntomas del paciente y los hallazgos clínicos importantes.	1
	**3c**	Los principales diagnósticos, intervenciones terapéuticas y resultados	1
	**3d**	Conclusión— ¿Cuáles son las principales lecciones que se pueden extraer de este caso?	1
**Introducción**	4	Breve resumen de los antecedentes de este caso haciendo referencia a la literatura médica pertinente.	1-2
**Información del paciente**	**5a**	Información demográfica (como edad, sexo, origen étnico, profesión).	2
	**5b**	Principales síntomas de paciente (sus principales molestias).	2
	**5c**	Historial médico, familiar y psicosocial que incluya la dieta, el estilo de vida y la información genética pertinente.	2
	**5d**	Enfermedades concomitantes pertinentes, incluyendo intervenciones anteriores y sus resultados	2
**Hallazgos clínicos**	6	Describir los hallazgos pertinentes de la exploración física (EF).	2
**Calendario**	**7**	Describa hitos importantes relacionados con sus diagnósticos e intervenciones (tabla o figura)	2, 4
**Evaluación diagnóstica**	8a	Métodos diagnósticos (como la EF, analíticas, técnicas de obtención de imágenes, cuestionarios).	2
	8b	Problemas para el diagnóstico (como económicos, lingüísticos o culturales).	2
	8c	Razonamiento diagnóstico, incluidos otros posibles diagnósticos tenidos en cuenta	2
	8d	Características de pronóstico (como los estadios en oncología) cuando proceda.	2
**Intervención terapéutica**	**9a**	Tipos de intervención (como farmacológica, quirúrgica, preventiva, autocuidados).	2
	**9b**	Administración de la intervención (como dosis, concentración, duración).	2
	**9c**	Cambios en la intervención (con justificación).	2
**Seguimiento y resultados**	10a	Resultados evaluados por el médico y por el paciente.	2
	10b	Resultados importantes de la prueba de seguimiento	2
	10c	Observancia de la intervención y tolerabilidad a la misma (¿cómo se ha evaluado?).	2
	10d	Acontecimientos adversos e imprevistos.	2
**Discusión**	**11a**	Puntos fuertes y limitaciones en el manejo de este caso.	3-4
	**11b**	Discusión de la literatura médica pertinente.	3-4
	**11c**	Justificación de las conclusiones (incluida la evaluación de las posibles causas)	3-4
	**11d**	Las principales lecciones que se pueden extraer de este informe de caso.	3-4
**Perspectiva del paciente**	12	¿Comunicó el paciente su perspectiva o experiencia? (Incluir siempre que sea posible).	
**Consentimiento informado**	**13**	¿Dio su consentimiento informado el paciente? Facilítelo si se le solicita.	**Sí** ☒ **No** ☐
